# Supplementary material for: Ready-to-use nanopore platform for the detection of any DNA/RNA oligo at attomole range using an Osmium tagged complementary probe
Source: Sci Rep. 2020 Nov 13;10:19790. doi: 10.1038/s41598-020-76667-1 (PMC7666163; doi:10.1038/s41598-020-76667-1)
Supplement: Supplementary file 1 — Supplementary Information. [file 41598_2020_76667_MOESM1_ESM.pdf]

Supplementary Information

Ready-to-use nanopore platform for the detection of any DNA/RNA oligo at attomole range using an Osmium tagged complementary probe.

Albert S. W. Kang[1], Janette G. Bernasconi[1], William Jack[2] and Anastassia Kanavarioti[1*]
[1]Yenos Analytical LLC, 4659 Golden Foothill Pkwy, Suite 101, El Dorado Hills, CA 95672, USA
[2]New England BioLabs, Inc., Ipswich, MA, USA
Correspondence should be addressed to A.K. (email: tessi.kanavarioti@gmail.com)

```
 _  _  _  _  _  _  _  _  _  _  _
 / \ / \ / \ / \ / \ / \ / \ / \ / \ / \ / \
( O | s | B | p | D | e | t | e | c | t )
 \_/ \_/ \_/ \_/ \_/ \_/ \_/ \_/ \_/ \_/ \_/
============ Thresholds ============
Event duration (in tps): 4 - 300
Lowest Ir/Io < 0.55
All Ir/Io < 0.6
=======================================
Processing channel 1...
400 events detected.
# Channel 1, Sampling rate: 3012.0 Hz, Io: 251.7634153366089 pA
```

1	353587	353611	0.06775701
2	398370	398375	0.11623832
3	407336	407348	0.08820094
4	412256	412438	0.21670561
5	447187	447197	0.07593458
6	453217	453249	0.06600467
7	492322	492371	0.07009346
8	493357	493361	0.32476636
9	497867	498043	0.21728972
10	520164	520179	0.19742991
11	542446	542455	0.06074766
12	567601	567612	0.08528037
13	595027	595031	0.33294393
14	650430	650488	0.08002336
15	650828	650858	0.16647196
16	742841	743058	0.04614486
17	792186	792318	0.12733645
18	832875	832913	0.04672897
19	841164	841188	0.28387851
20	907859	907868	0.27686916
21	1073757	1073771	0.19801402
22	1075537	1075541	0.34521028
23	1080276	1080460	0.15887851
24	1081066	1081078	0.15478972
25	1101348	1101364	0.21378505
26	1110665	1110672	0.26109813
27	1132460	1132678	0.23948598
28	1178961	1179019	0.39252336
29	1211889	1211901	0.26518692
30	1267382	1267386	0.28679907

```
 _  _  _  _  _  _  _  _  _  _  _
 / \ / \ / \ / \ / \ / \ / \ / \ / \ / \ / \
( O | s | B | p | D | e | t | e | c | t )
 \_/ \_/ \_/ \_/ \_/ \_/ \_/ \_/ \_/ \_/ \_/
============ Thresholds ============
Event duration (in tps): 4 - 300
Lowest Ir/Io < 0.55
All Ir/Io < 0.6
=======================================
Processing channel 1...
Processing channel 2...
Processing channel 3...
Processing channel 4...
39 events detected.
# Channel 4, Sampling rate: 3012.0 Hz, Io: 240.23457124829292 p[illegible]
```

1	567674	567705	0.09669523
2	766999	767209	0.29681763
3	918896	918925	0.09669523
4	1050173	1050204	0.30966952
5	1599517	1599525	0.20379437
6	1680746	1680758	0.28580171
7	2049151	2049162	0.16034272
8	2051214	2051247	0.18665851
9	2087063	2087068	0.18359853
10	2297779	2297785	0.15422277
11	2544443	2544458	0.26376989
12	2862924	2862940	0.28335373
13	3044089	3044100	0.41003672
14	3150965	3150971	0.35495716
15	4810901	4810911	0.41554468
16	4810943	4810950	0.41615667
17	4811778	4811783	0.48347613
18	4812081	4812139	0.41432069
19	4812197	4812203	0.46511628
20	5915973	5915996	0.43329253
21	5916909	5916964	0.41432069
22	6204350	6204361	0.29620563
23	6218284	6218451	0.13219094
24	6314244	6314248	0.33659731
25	6533152	6533163	0.24235006
26	6694009	6694020	0.34210526
27	6716411	6716419	0.19828641
28	7095854	7095865	0.43941249
29	7136165	7136282	0.45777234
30	7214510	7214528	0.18849449

Figure S1: Samples of *tsv* files, obtained by running the *OsBp_detect* software on *fast-5* files. Left, sample probe T8(RNA); right, sample is a mixture of d(CT)$_{10}$:T8(RNA)=1:1, both in about 90% ONT buffer (see caption of Fig. 2a for experimental conditions).

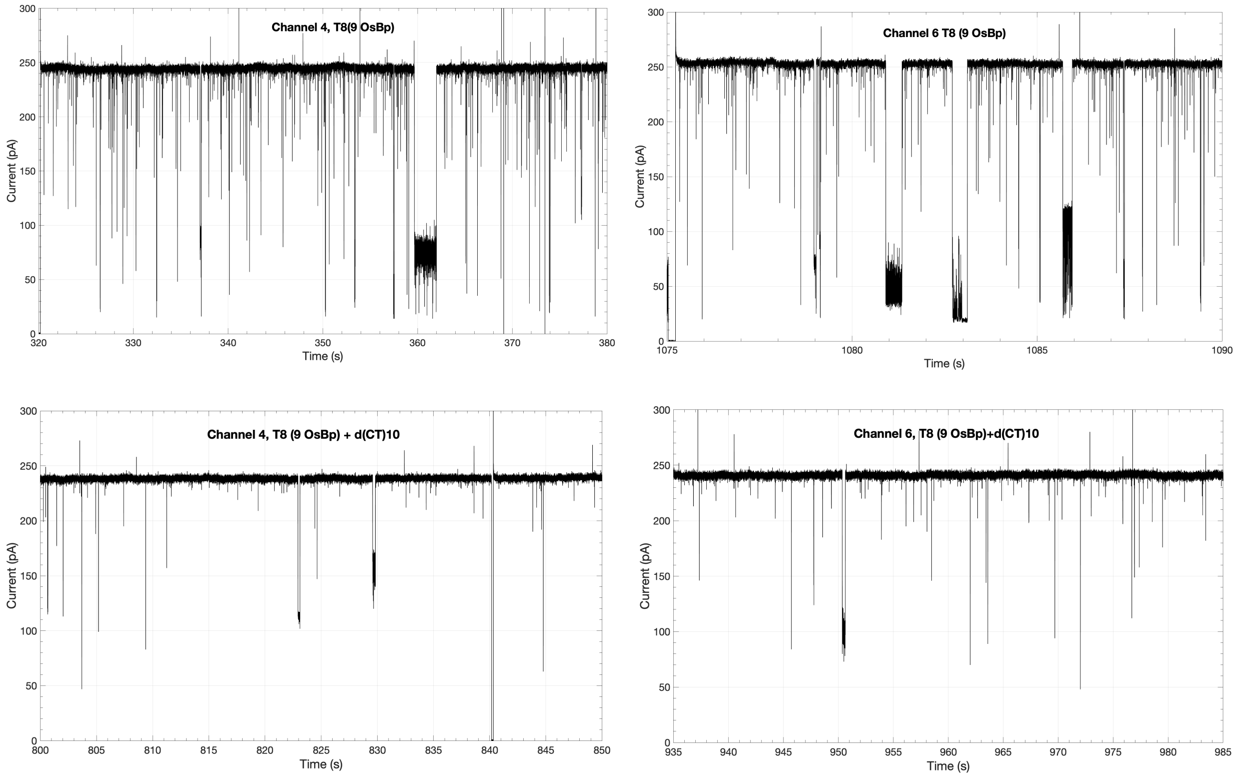

Figure S2: *I-t* recordings from two nanopore experiments ranging from 15 to 60s. Top, probe T8(RNA), *i-t* recordings from two different channels. Bottom, mixture of d(CT)$_{10}$: T8(RNA)=1:1, *i-t* recordings from the same two channels as on the top. Vertical lines that cross the x-axis (=0 pA) are instrument generated lines by voltage reversal, and not events. Top recordings show multiple and deep events, bottom recordings show few and shallow events. Shallow events are attributed to molecules bumping at the pore aperture, without traversing the pore and they are not counted when selecting “All Ir/Io < 0.6” (see Fig. S1, and Experimental Section).

Table S1: Osmylation of single-stranded M13mp18 inhibits DNA polymerase extension[1]

CE observed R(312/272)[2]	% Osmylated pyrimidines[3]	Background-corrected cpm	
		Klenow exo-	Klenow
0	0	66669	35921
0.14	12	1111	800
0.26	22	429	366
0.45	38	341	327
0.94	80	145	65

[1] DNA polymerase extension was primed with a 5-fold molar excess of two annealed primers, 5'TAAAGTTTTGTCGTCTTTCC3' and 5'GCGCTCACTGCCCGCTTTCC3'. Final reactions contained 40 µg/mL osmylated circular ssM13mp18 from Bayou Labs, 84 nM each of the above primers, 0.2 mM dATP, dGTP, dTTP, 0.02 mM [✓-^{32}P]-dCTP, 1X NEBuffer 2.1, and 40 U/mL of Klenow or Klenow exo- DNA Polymerase. Reactions were incubated at 37°C for 10 minutes, and nucleotide incorporation determined by an acid precipitation assay.

[2] Observed R(312/272) is the observed Absorbance at 312 nm divided by the observed absorbance at 272 nm from Capillary Electrophoresis (CE) analysis of partially osmylated circular ssM13mp18 from Bayou Labs.

[3] Calculated from CE observed R(312/272) divided by the value of R(312/272)=1.18 at reaction completion, i.e. when all the pyrimidines carry one OsBp moiety (reference 54).

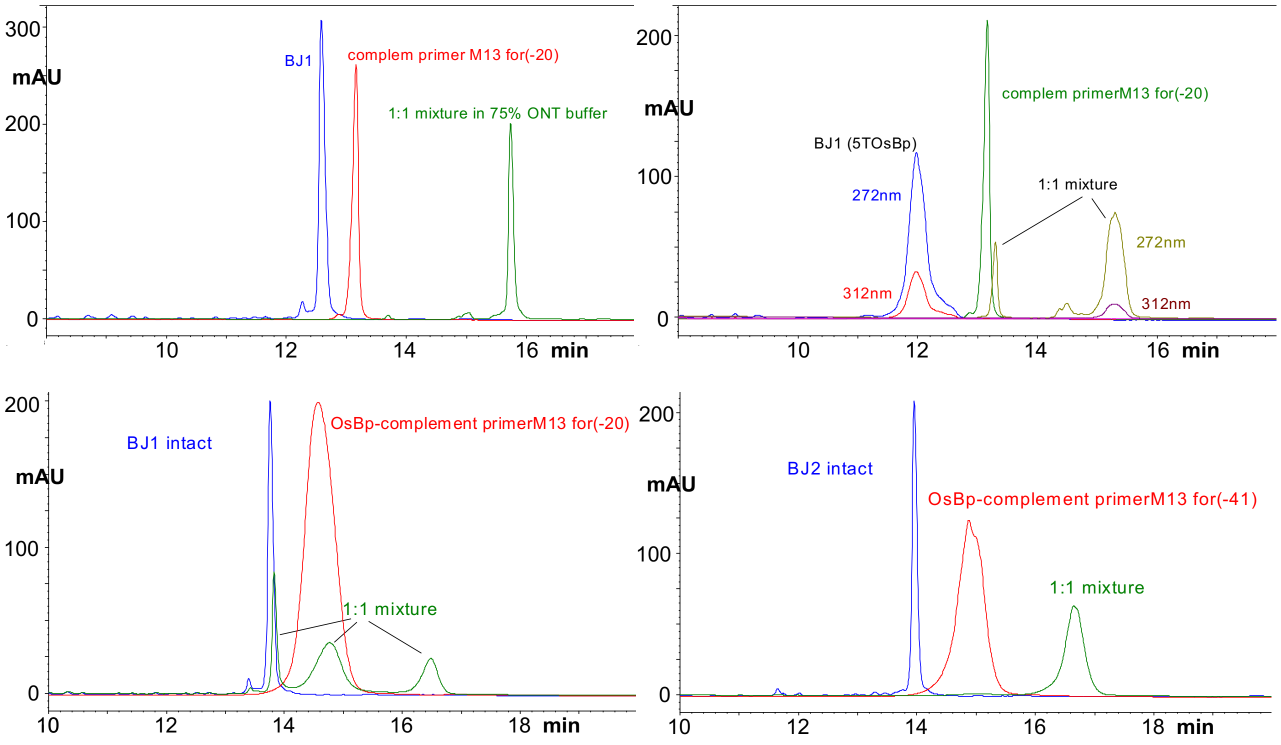

Figure S3: HPLC profiles of individual components and their 1:1 mixtures in a sample solvent about 90% ONT buffer. All HPLC profiles shown at 260 nm, with the exception of the osmylated probe and hybrid in the top right profile that is shown at 272 nm and 312 nm. Hybridization is evident by a mixture HPLC profile where the main peak elutes after the individual components. Top left, hybridization shown for two intact oligos, BJ1 and its complementary intact oligo (complement primerM13*for*(-20)). See Fig. 3b for comparable result with another pair of intact oligos. Top right, hybridization shown for probe BJ1 with 5 OsBp out of 30nt, and its complementary intact oligo (complement primerM13*for*(-20)). Left bottom, only partial hybridization is shown for intact BJ1 and its complementary osmylated complement primerM13*for*(-20) with 11 OsBp out of 35nt. Right bottom, hybridization is shown for intact BJ2 and its complementary osmylated complement primerM13*for*(-41) with 6 OsBp out of 35nt. HPLC method B is used for analysis (see Experimental Section).

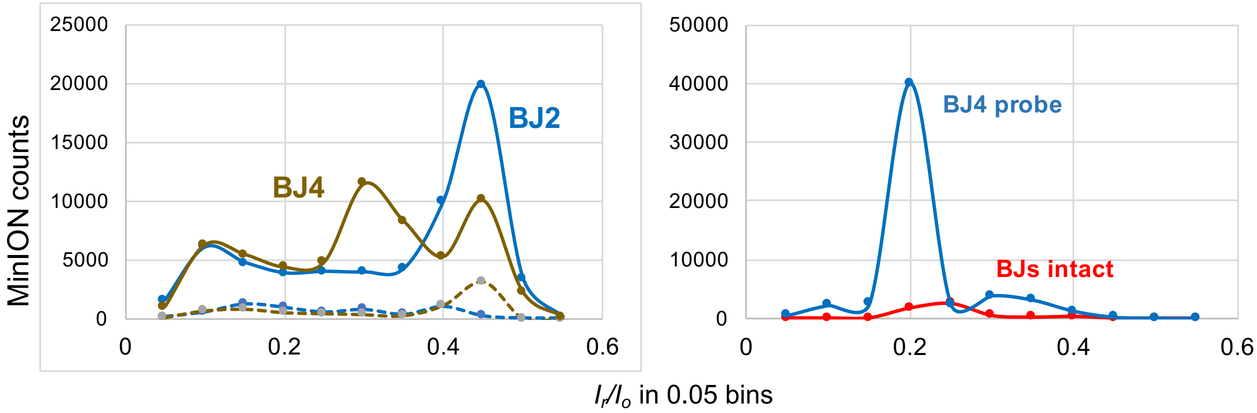

Figure S4: **Left, Nanopore experiments with probes BJ2 and BJ4 show few events at -180mV and numerous events at -220mV.** Probes BJ2, blue traces, and BJ4, brown traces, tested at -180mV (dashed traces) show negligible number of counts. No new sample was added, the voltage was raised to -220mV and probes were tested at -220 mV (solid traces) and numerous events were detected**.** Both probe samples were used at a 0.2nmole load. Experiments were conducted on the same flow cell in the order BJ2 at -180mV, BJ2 at -220mV, BJ4 at -180mV and BJ4 at -220mV; the duration of each experiment was 1h. Data acquisition and analysis as described under Fig. 2a (see text).The dramatic difference in counts at the different applied voltage clearly suggests that these probes and other probes, of similar design, do not traverse the proprietary CsGg nanopore at the lower voltage and require high applied voltage of about -220mV to translocate.

Right, Consecutive nanopore experiments using the same device, the same duration, and voltage. First, we tested a sample made of equimolar amounts of the intact BJ1, BJ2, BJ3, BJ4 (BJs) at a total concentration of 5μM, and then we tested a sample of the osmylated probe BJ4 also at 5 μM. The counts produced by the intact DNA oligos are 1/10 of the counts produced by the DNA probe BJ4 under identical conditions. We attribute the different translocation profiles observed for probe BJ4 to an aged flow cell (left) and to a new flow cell (right).

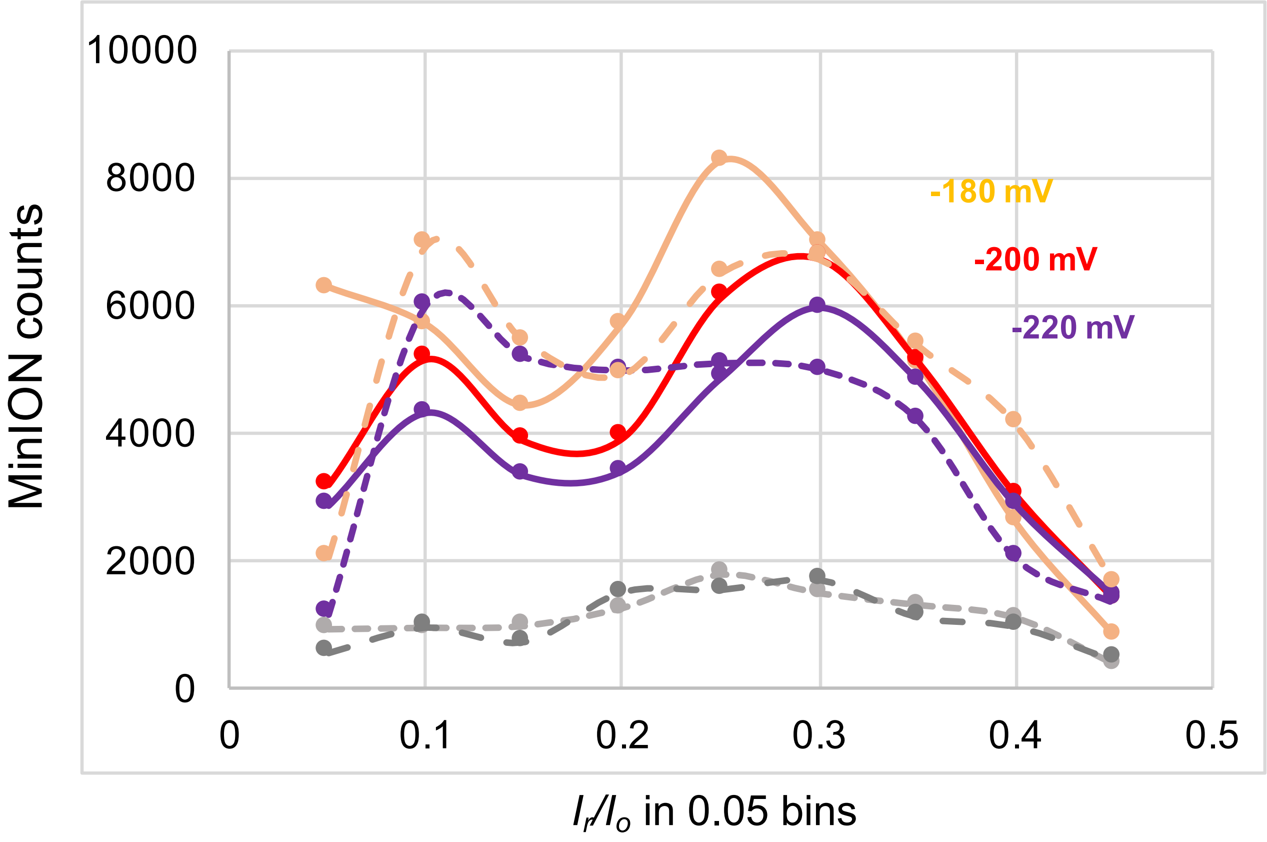

Figure S5: Effect of applied voltage on the counts observed with a mixture of intact miRNA122 and miRNA140 at 10µM each at -180mV (orange trace), at -200mV (red trace), at -220mV (purple trace). Data shown at -180mV (dashed orange trace), are the same data as in 2b (Flongle, red trace)) but normalized by multiplying with 10, as the MinION has about 10 times more working channels compared to the Flongles that we purchased/used. Increased applied voltage reduces slightly the count of events, consistent with faster translocation and reduced detectability. An experiment with 10µM of miRNA21-A_{15} at -220mV (dashed purple line) exhibits comparable counts with the combo of miRNA122 and miRNA140, but a distinct profile compared to the miRNA with no A_{15}-tail. The effect of voltage on the intact RNAs is in stark contrast to the effect of voltage on most of the probes tested in this study. No detectable difference in counts is observed with the control/buffer between -200 and -220mV (light and dark dashed grey traces, respectively).

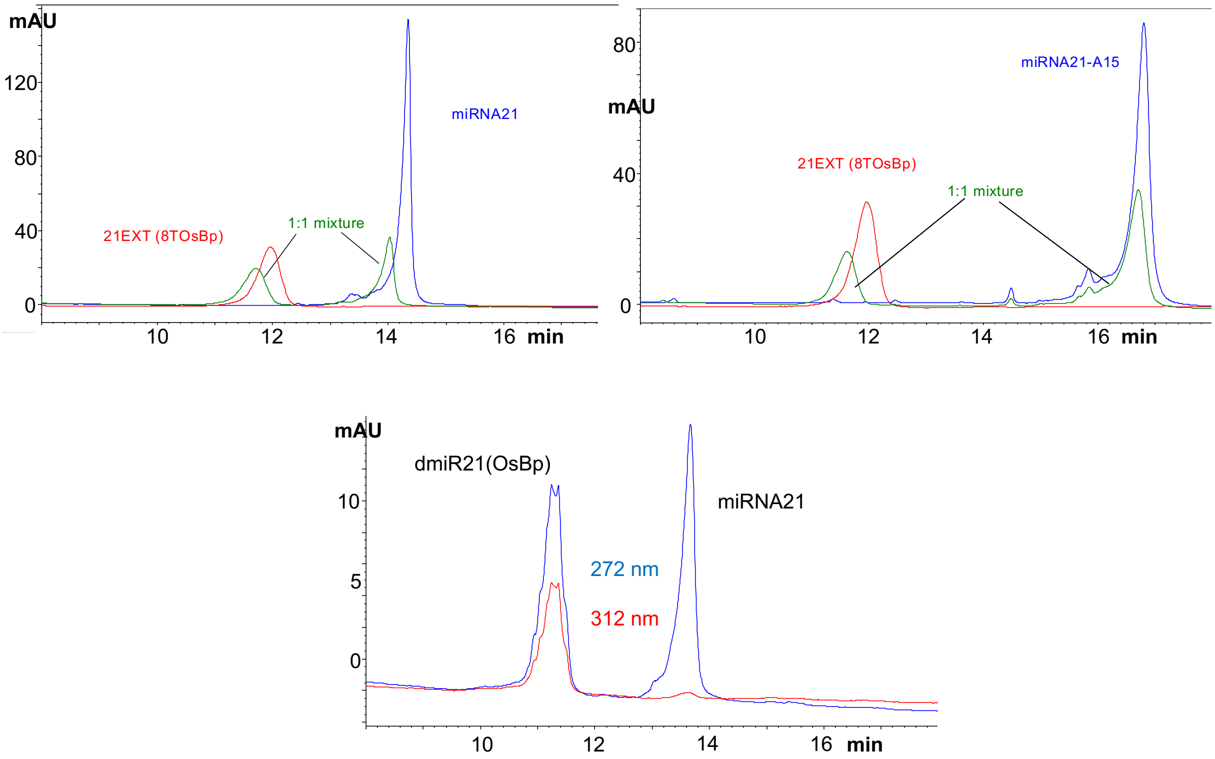

Figure S6: HPLC profiles of 1:1 mixtures (non-hybrids) of miRNA21 with probe dmiR21 (with 6 T(OsBp), or miRNA21 and miRNA21-A15 using probe 21EXT (with 8 T(OsBp), see sequences in table). Left profile the same as in Figure 3c, repeated here for direct comparison with the right profile. The difference in these two cases is that, due to the added A_{15}-tail, miRNA21-A_{15} elutes couple of min later compared to miRNA21.

Bottom profile, one sample only, the 1:1 mixture of miRNA 21 with dmiR21 at two different wavelengths to show that only the probe exhibits absorbance at 312nm, whereas native nucleic acids, like miRNA21, exhibit negligible absorbance at 312nm. Two HPLC peaks are consistent with no hybridization.

Samples in about 90% ONT buffer as the sample solvent. HPLC profiles obtained with HPLC method B (Experimental Section). HPLC profile of the mixture sample matches closely the sum of the HPLC profiles of the two components, providing evidence for no detectable hybridization in these two cases.

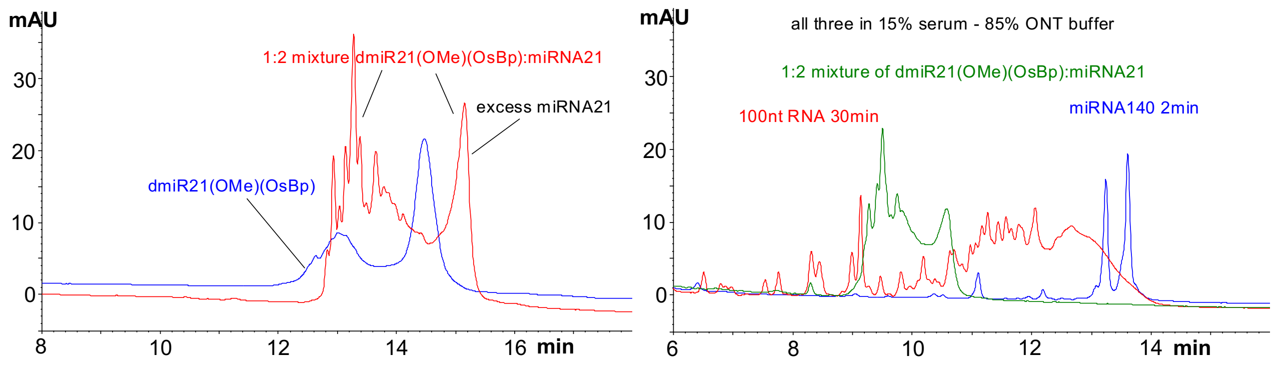

Figure S7: Stability of hybrid in 15% serum -85% ONT buffer. Left, repeat of Fig. 7a (red trace) in order to compare directly with HPLC profile (green trace) to the right. Right: HPLC profiles of three samples in 15% serum -85% ONT buffer: miRNA140 (blue trace) 2min incubation before analysis, 100nt RNA (red trace) 30min incubation before analysis. The longer incubation is why the degradation of 100nt RNA appears more severe compared to the degradation of miRNA140. Mixture of dmiR21(OMe)(OsBp): miRNA21=1:2 in about 5% water- 95% ONT buffer (left, red trace) and the same mixture in 15% serum -85% ONT buffer (right, green trace). The HPLC profiles (red trace in left profile and green trace in right profile) appear comparable suggesting that the hybrid suffers insignificant degradation in 15% serum - 85% ONT buffer. HPLC method B used for these analyses (see Fig. S10 and Experimental Section).

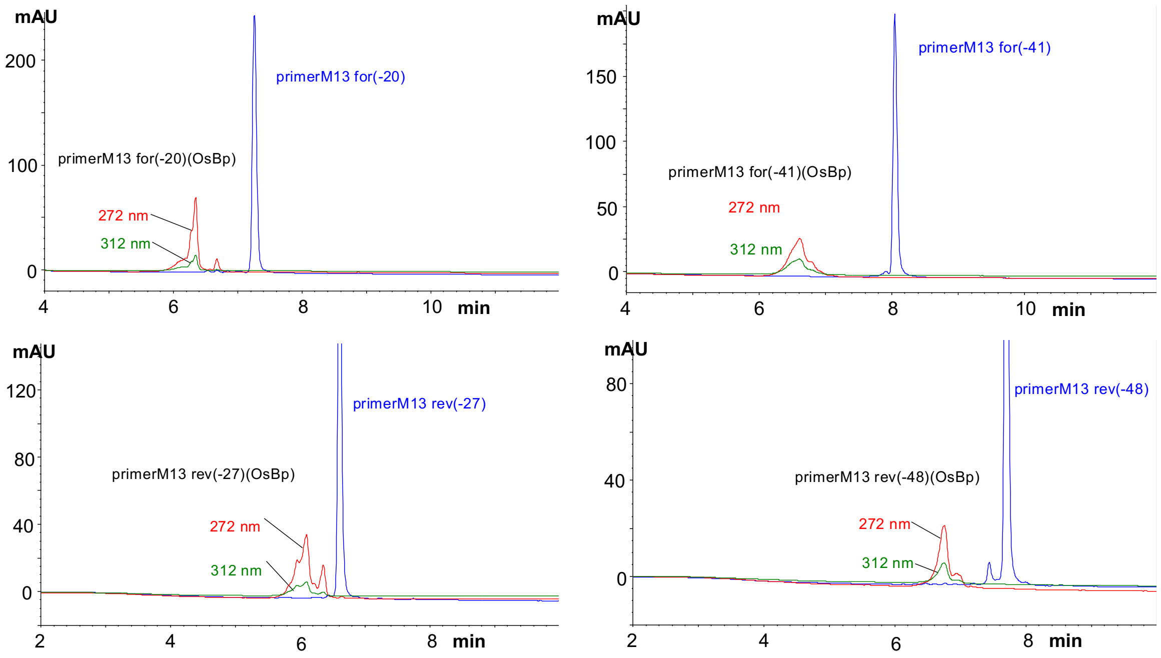

Figure S8: HPLC profiles of the four intact M13 primers and their corresponding T-osmylated derivatives, using HPLC method A (Experimental Section). Analysis of oligos in water as the sample solvent. T-osmylation of these oligos was conducted using protocol o (see Experimental Section). The reason osmylated oligos appear as multiple peaks is because top or bottom addition of OsBp to the C5-C6 double bond leads to topoisomers (see text), that this chromatography resolves.

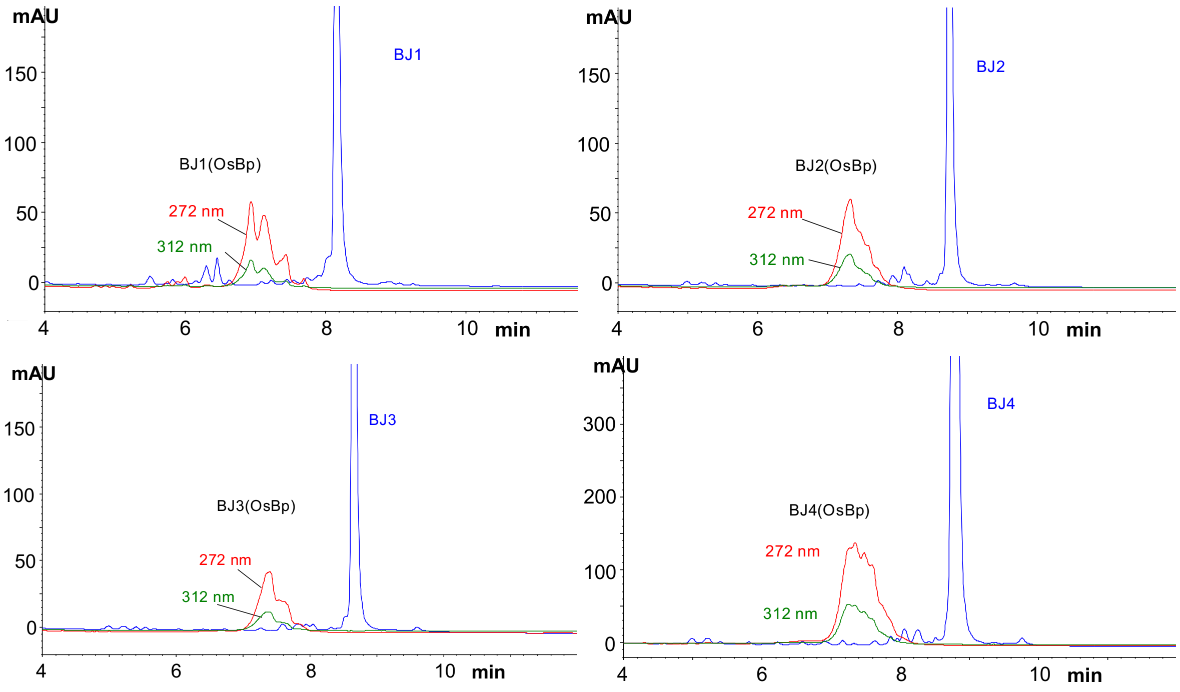

Figure S9: HPLC profiles of the four intact BJ1-4 and their corresponding T-osmylated derivatives using HPLC method A. Analysis of oligos in water as the sample solvent. T-osmylation of these oligos was conducted using protocol o (see Experimental Section).

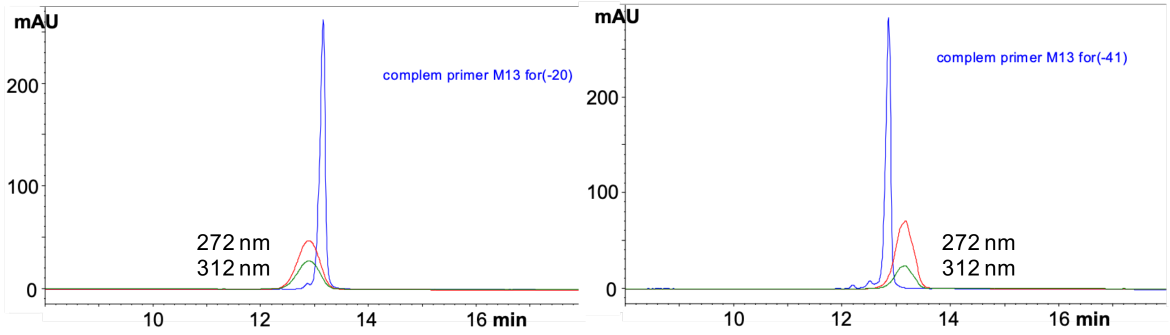

Figure S10: HPLC profiles of the 2 intact complements of primerM13*for*(-20) and primerM13*for*(-41) shown at 260 nm, and their corresponding T-osmylated derivatives shown at 272nm and 312nm using HPLC method B (see Experimental Section). Analysis of oligos in water as the sample solvent. Briefly HPLC Method B is using the DNA Pac PA200 HPLC column from ThermoFisher Scientific at the 2x250mm configuration with 0.45mL/min flow and 15ºC column compartment. Solvents are aqueous pH 8.0±0.2 mobile phases A (MPA) and mobile phase B (MPB) with 25mM TRIS.HCL buffer; MPB is 1.5 M NaCl. Initial conditions are 90% MPA – 10% MPB, and the gradient is from 10% to 50% MPB in 20 min. The total analysis time including column equilibration is 30min. T-osmylation of these oligos was conducted using protocol o (see Experimental Section). Right profile shows an atypical, but confirmed result, namely an osmylated conjugate that elutes later compared to the parent intact nucleic acid.

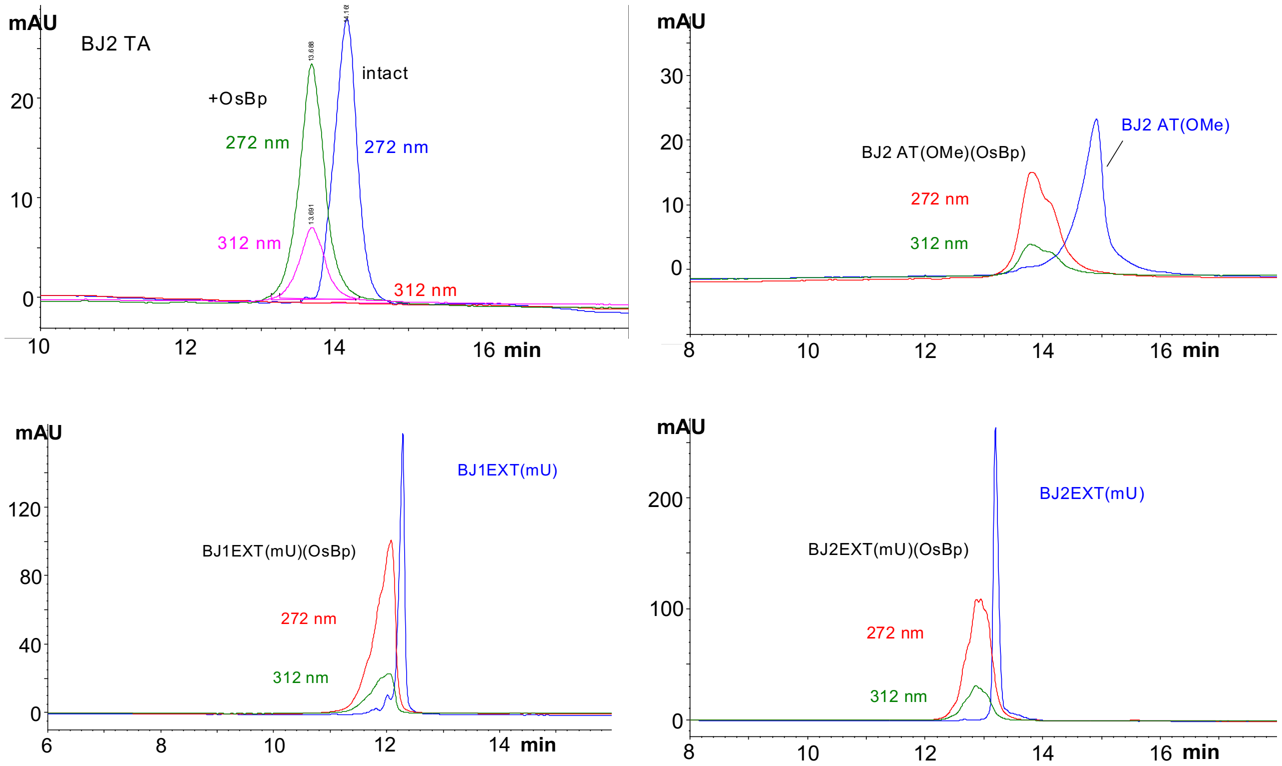

Figure S11: HPLC profiles of advanced probe designs and their osmylated derivatives. BJ2 TA(OMe) (top, left) and BJ2 AT(OMe) (top, right), as well as BJ1EXT(mU) (bottom, left) and BJ2EXT(mU) (bottom, right) shown at 260nm and their corresponding T-osmylated derivatives shown at 272nm and 312nm (see sequences in table in text). HPLC profiles obtained with HPLC method B (see Fig. S10, and Experimental Section). Materials in water as sample solvent. A nanopore experiment conducted with probe BJ2 TA(OMe) indicated excellent translocation properties with numerous counts at a relatively low probe load (Fig. 4d). A nanopore experiment conducted with BJ2 AT(OMe) at comparable conditions was not conclusive. Since the only difference between the two probes is that the 3'-end on one is the 5'-end on the other, it is not clear whether or not BJ2 TA(OMe) with the Ts at the 5'-end and the As at the 3'-end is a superior probe compared to BJ2 AT(OMe) with the Ts at the 3'-end and the As at the 5'-end.

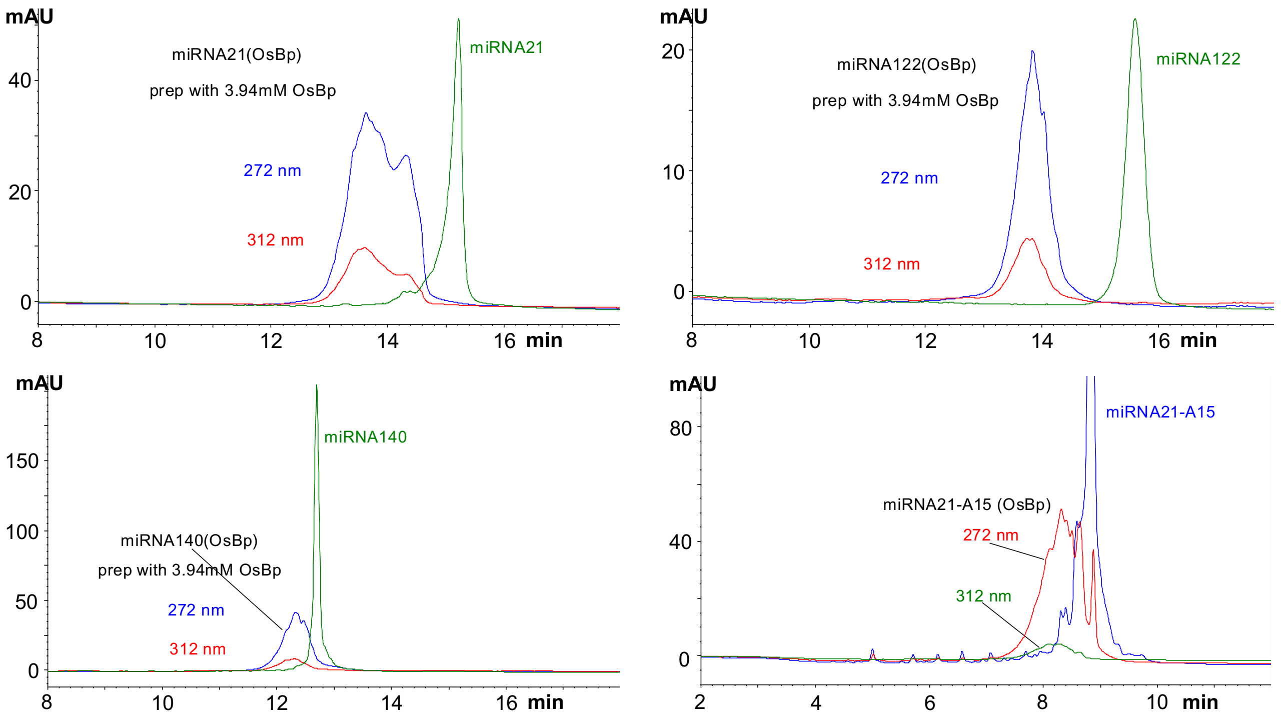

Figure S12: HPLC profiles of the intact miRNA21, miRNA122, miRNA140 and miRNA21-A_{15} (these are the -5p sequences) shown at 260nm and their corresponding partially osmylated derivatives; different materials are analyzed at different sample load. Osmylation protocol o was used for miRNA21-A_{15} and protocol c for the other 3 miRNAs (see table and Experimental Section). HPLC method A used for the analysis of miRNA21-A15 and HPLC method B for analysis of the other 3 miRNAs (see Experimental Section). Materials in water as the sample solvent.

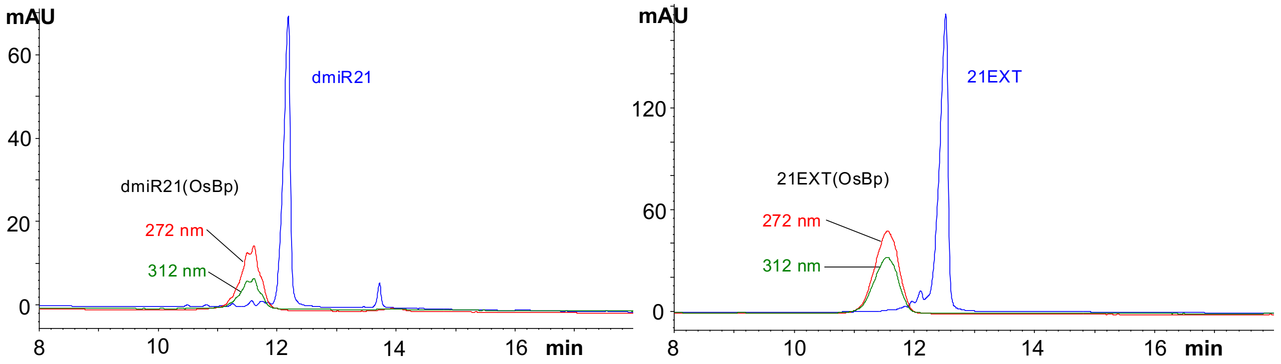

Figure S13: HPLC profiles of unsuccessful probes for miRNA21. Left, HPLC profiles of intact dmiR21 at 260nm and the T-osmylation product at 272nm and 312nm. Right, HPLC profiles of intact 21EXT and its T-osmylation product. Osmylation was carried out using protocol o 40min with 2.63mM OsBp, earlier process where bipy was dissolved after adding OsO_4 (see Experimental Section). Materials in water for analysis, and analysis was done using HPLC method B (see Fig. S10 and Experimental Section).

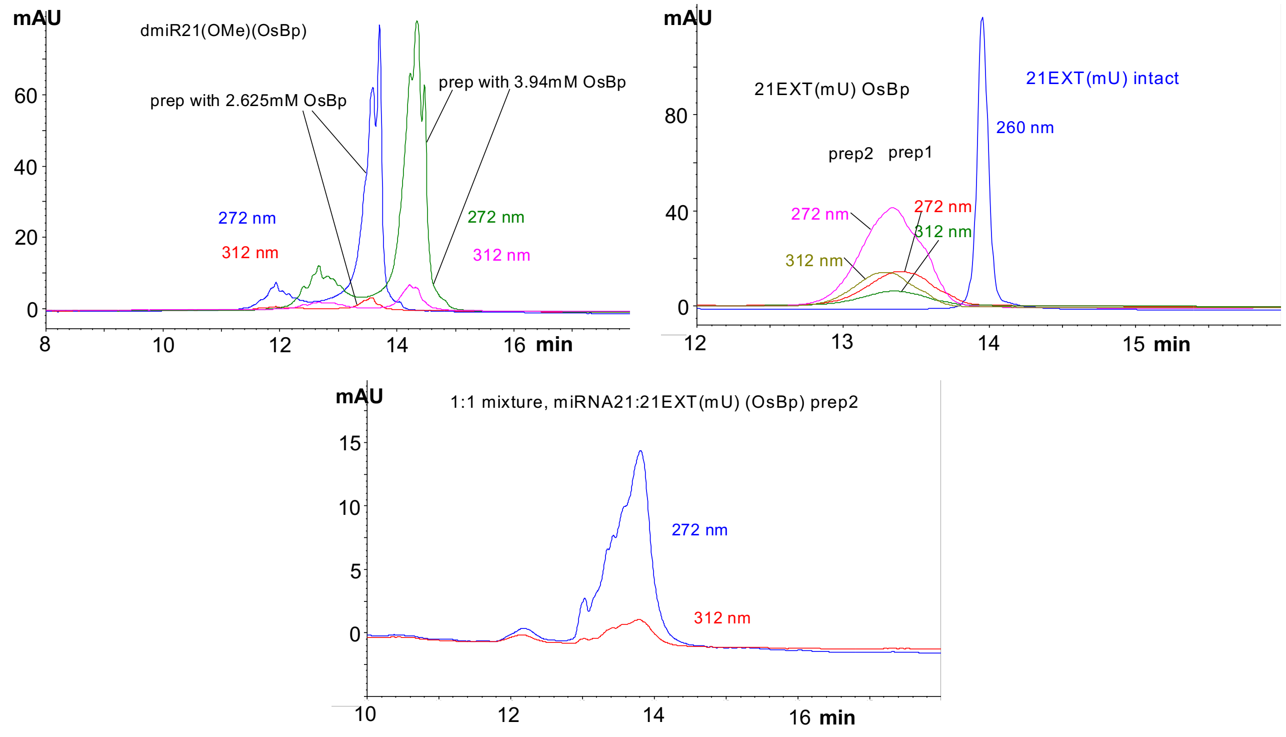

Figure S14: **HPLC profiles of successful probes for miRNA21.** Left, HPLC profiles of the osmylation products of dmiR21(OMe) using protocol b 30min with 2.63mM OsBp and protocol c 30min with 3.94mM OsBp. A third protocol (d) with 30min incubation using 5.25mM OsBp was used to osmylate this probe for the nanopore experiment (Fig. 6b). This was necessary because dmiR21(OMe) contains no Ts, and using protocols b and c results in very low levels of osmylation and diminished detectability. Right, probe 21EXT(mU) intact and its osmylation products with the two different protocols; prep1, using protocol a 45min with 2.63mM OsBp and prep2 protocol b 30min with 2.63mM OsBp. Bottom profile, repeat of Fig. 7c in order to compare the HPLC profile of the hybrid with the HPLC profile of the probe alone (b, above) and see that they are distinct. Specifically using the same HPLC method, probe 21EXT(mU)OsBp prep2 exhibits a peak at about 13.3 min (right figure), whereas the hybrid, i.e. 1:1 mixture of miRNA21 with this probe exhibits a peak at about 13.8 min (bottom figure). Analysis was done using HPLC method B (see Fig. S10 and Experimental Section).

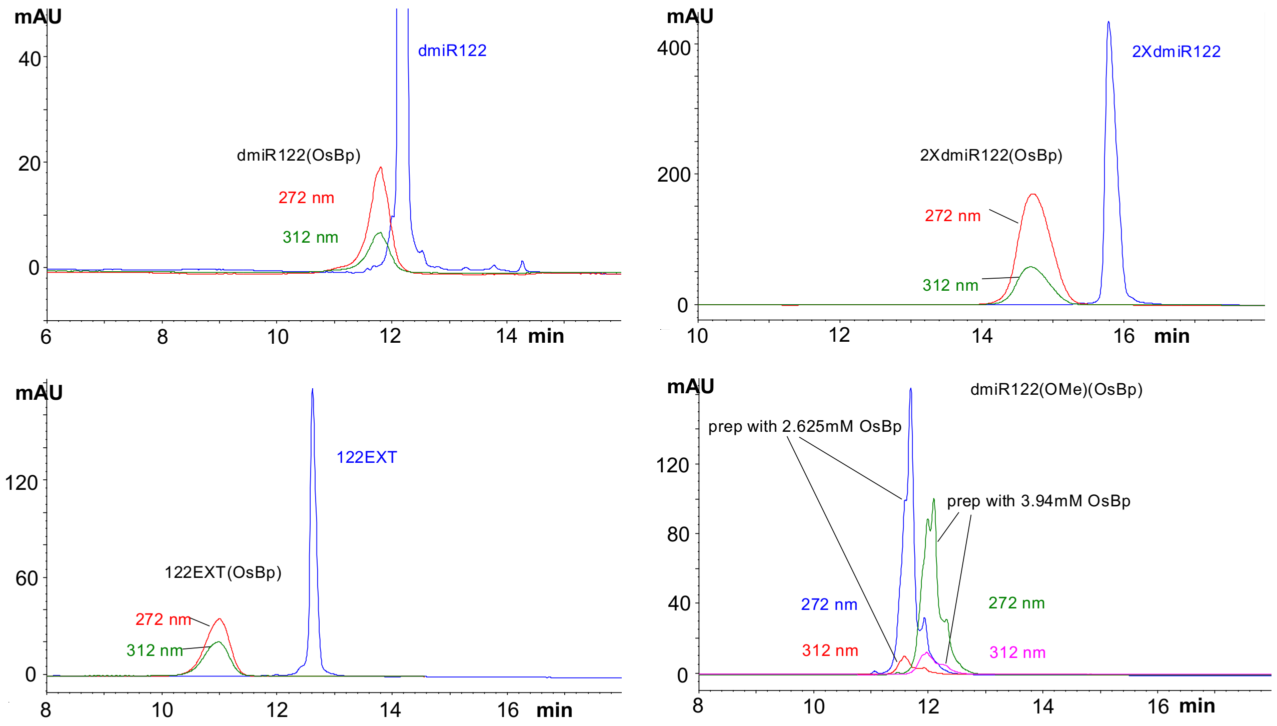

Figure S15: HPLC profiles of successful probes for miRNA122. HPLC profiles of the intact miRNA122 probes shown at 260nm and their corresponding T-osmylated derivatives (the actual probes) at 272 and 312nm. Osmylation protocol o was used to osmylate dmiR122, 2XdmiR122 and 122EXT. dmiR122(OMe) does not have any T, and was osmylated using protocols b or c (see table for sequences and for protocols). Materials were in water for analysis and analysis was done using HPLC method B (see Fig. S10 and Experimental Section).

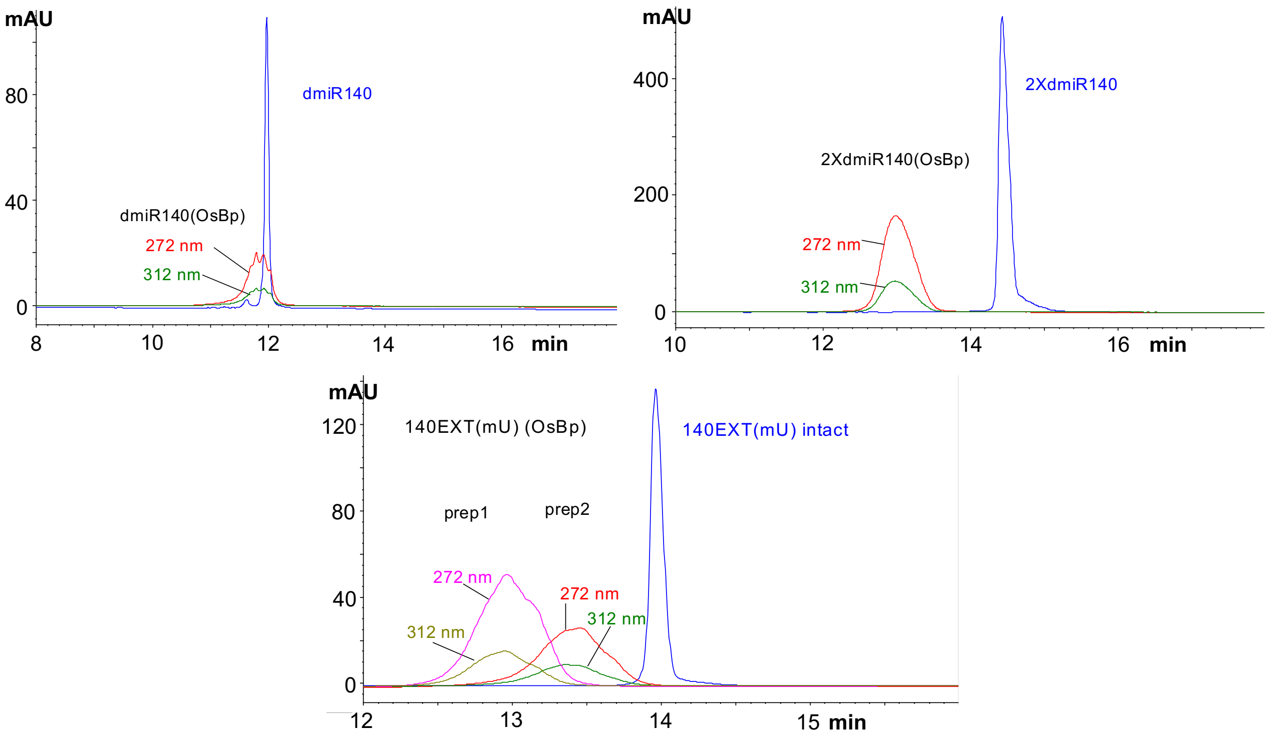

Figure S16: HPLC profiles of the intact miRNA140 probes shown at 260nm and their corresponding osmylated derivatives (the actual probes) shown at 272nm and 312nm. Top HPLC profiles with dmiR140 (left) and 2XdmiR140 (right) using osmylation protocol o, 40min with 2.63mM OsBp (earlier process, bipy not dissolved prior to OsO_4 addition. Bottom, probe 140EXT(mU) intact and its osmylation products with the two different protocols; prep2, using protocol a, 45min with 2.63mM OsBp, and prep1 protocol b, 30min with 2.63mM OsBp. Materials in water for analysis, and analysis was done using HPLC method B (see Fig. S10 and Experimental Section).

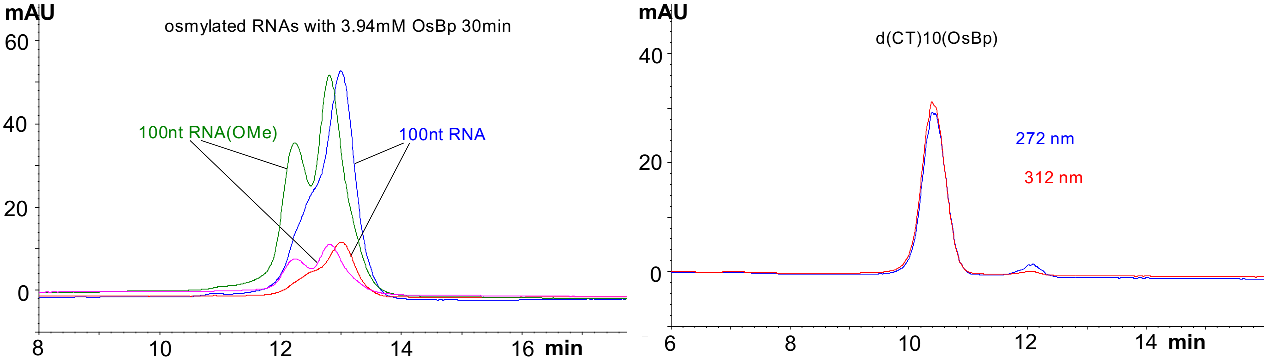

Figure S17: HPLC profiles of partially osmylated RNAs and DNA. Left, HPLC profiles of partially osmylated 100nt RNA and 100nt RNA(OMe) using osmylation protocol c (see table and Experimental Section). Right, HPLC profiles of T-osmylated d(CT)$_{10}$ using protocol b. Materials in water as the sample solvent. HPLC method B was used for all the samples and HPLC profiles are shown at 272nm and 312nm. Osmylation protocols and HPLC method can be found in the Experimental Section.

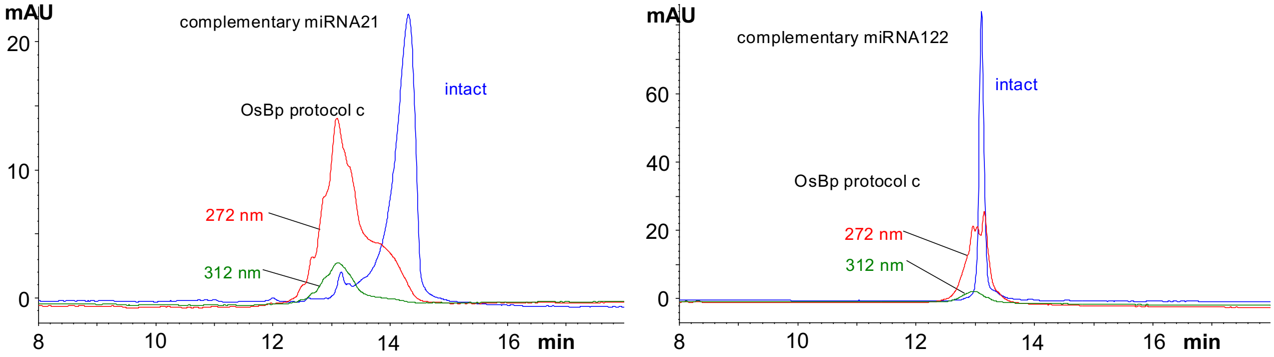

Figure S18: HPLC profiles of partially osmylated 22nt RNAs. Left, complementary miRNA21 and Right, complementary miRNA122. Osmylation protocol c was used, 30min with 3.94mM OsBp.

Table S2: Entries obtained from table in the text: Osmylation protocol c (30min with 3.94mM OsBp)

Oligo ID	No of U in the sequence	No of Py(OsBp) for oligos with native U	No of Py(OsBp) for oligos with modified U	comment
miRNA21	8	2.950		
Complement of miRNA21, RNA	6	2.013		
miRNA122	7	3.040		
dmiR21(OMe)	6		1.365	All bases 2'-OMe
Complement of miRNA122, RNA	4	1.573		
dmiR122(OMe)	4		1.65	All bases 2'-OMe
100nt RNA	27	11.96		
100nt RNA(OMe)			11.19	50% of bases 2'-OMe

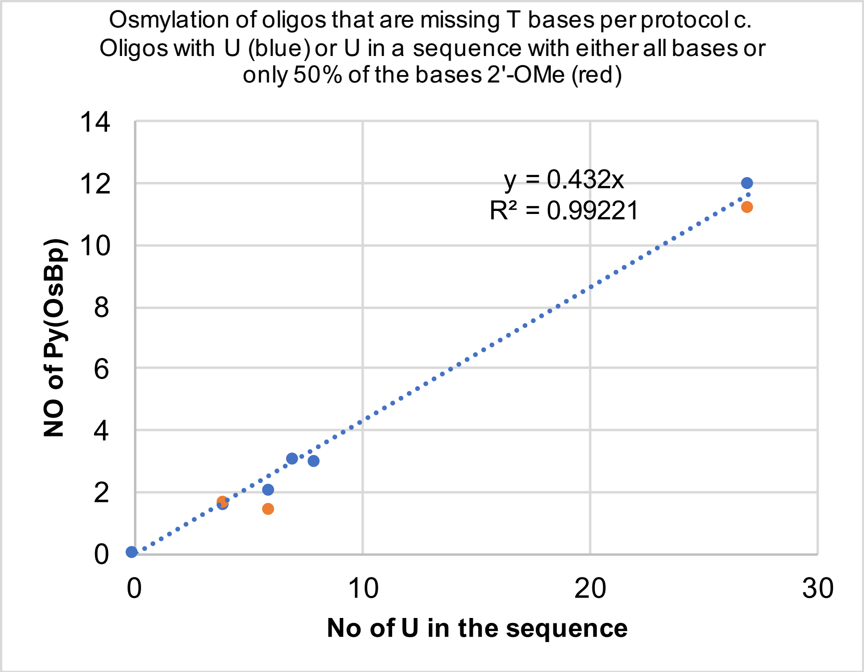

Figure S19: Graph using the data from Table S1 above. A good linear correlation is obtained for the number of osmylated pyrimidines in an oligo, which is missing Ts, as a function of the number of U in a sequence. The correlation does not appear to depend heavily on whether or not the sequence is a DNA, RNA or carries 2'-OMe groups on all or on a portion of the bases. The linear correlation may be used to estimate the number of osmylated pyrimidines per protocol c for any given sequence. The linear correlation is attributed to the observation that U is osmylated 4.7-times faster compared to C[44], and to the conditions of protocol c that yield only a small percentage of osmylated pyrimidines, and not a practically 100% osmylated oligo.
